#  Capturing growth indices on the road to health booklets in clinics in Free State, South Africa

**DOI:** 10.4102/hsag.v29i0.2587

**Published:** 2024-07-12

**Authors:** Patience O. Legoale, Mashudu Manafe

**Affiliations:** 1 Department of Human Nutrition and Dietetics, School of Health Care Sciences, Sefako Makgatho Health Sciences University, Pretoria, South Africa; 2 Department of Health, Provincial Office, Mangaung, South Africa

**Keywords:** growth monitoring, road to health booklet, anthropometric assessment of children under 5 years of age, growth indices, malnutrition, nutritional status

## Abstract

**Background:**

Growth monitoring plays an essential role in the development of young children. Anthropometric indices are of utmost importance for healthcare professionals to identify children at risk of inadequate growth and malnutrition.

**Aim:**

This study aimed to assess the capturing of the growth indices in the Road to Health Booklets (RTHB) in clinics.

**Setting:**

The study was carried out in Mangaung Metropolitan municipal clinics in the Free State province, South Africa.

**Methods:**

A descriptive quantitative study was conducted using a checklist to audit 264 RTHBs. Descriptive statistics were used to analyse data.

**Results:**

The findings showed that birth weight was recorded in most 99% (*n* = 262) of the RTHBs. The mid-upper arm circumference (MUAC) was not recorded in 58% (*n* = 153) of the cases during the last visit. Weight-for-Age (WfA) was routinely plotted in 91% (*n* = 241) of the RTHB. The length or Height-for-Age (LHfA) was plotted in 38% (*n* = 99) of the RTHB and Weight-for-Length or height (WfLH) was plotted in 31% (*n* = 81) of the RTHB.

**Conclusion:**

The results demonstrated that certain anthropometric measures including MUAC, length, or height were absent from the records of the RTHB. Consequently, RTHB may not be effectively used as a means of evaluating nutritional status, affecting early detection of malnutrition in children.

**Contribution:**

The research makes a valuable addition to the existing body of knowledge for monitoring growth and measurement of anthropometric indices in the RTHB, as well as the appropriate execution of these practices.

## Introduction

Growth monitoring (GM) is of utmost importance in evaluating child growth and development, and represents one of the strategies that have been devised to combat malnutrition (Mabesa, Knight & Nkwanyana 2022). According to the United Nations Children’s Fund (UNICEF), GM serves as a preventive measure by facilitating communication and interaction with caregivers and generating appropriate actions that promote child growth (UNICEF 2014). Furthermore, GM is intended to serve as a diagnostic tool for health and nutrition surveillance and to prompt effective action in response to growth stalling. South Africa uses the Road to Health Booklet (RTHB), which is used to track a child’s development. Similar books in other countries help to evaluate their progress towards achieving the Sustainable Development Goals (SDGs) (Ferreira 2020). The most crucial SDG, SDG 2, focusses on eradicating hunger, attaining food security, and improving nutrition, and is monitored through indicators such as undernutrition, wasting, stunting, and obesity in children (Amaha 2020).

Most of deaths among undernourished children occur in the 64 countries classified as low-and middle-income countries (LMICs), with the highest prevalence observed in Asia and Africa (UNICEF, WHO & World Bank 2020). These deaths can be prevented through proper growth monitoring among others. Consequently, it is imperative for healthcare professionals (HCPs) to implement growth monitoring through the measurement and documentation of children’s anthropometric indicators, such as weight, length, height, and mid-upper-arm circumference (MUAC). The classification of nutritional status occurs when anthropometric measurements are obtained and plotted on linear graphs (Department of Health Guideline 2012). These anthropometric indicators are incorporated into the RTHB and adhere to the 2006 World Health Organization Child Growth Standards (WHO-CGS) to identify malnutrition and decrease the number of deaths associated with undernutrition in children under the age of five. In a study conducted by Massyn et al. (2020) in Mangaung, South Africa, it was reported that malnutrition ranked fourth out of ten causes of death among boys and girls under the age of five between 2012 and 2017.

To assess the nutritional status of young children, health workers record measurements for weight (at least monthly from birth), length (every 6 months from the age of 6 months), and MUAC (every 3 months from the age of 6 months) (Department of Health Guideline 2012). Additionally, these anthropometric measurements are plotted on weight-for-age (WfA), length or height-for-age (LHfA), and weight-for-length or height (WfLH) graphs to monitor growth trends. These trends provide a better understanding of the nutritional status of children and facilitate the timely implementation of interventions (Department of Health Guideline 2012).

A WfA value of (-2SD) indicates that a child is moderately underweight, while an LHfA value of ≤ 2SD indicates stunting (WHO & UNICEF 2009). Stunting arises from chronic malnutrition that is associated with long-term factors such as inadequate protein-energy intake, frequent infections, persistent poor feeding practices, and specific micronutrient deficiencies, particularly iron and zinc (Abu-Manga et al. 2021). Without adequate documentation of anthropometric measurements, malnutrition will continue to burden communities. Healthcare programmes should improve the training of health workers in the collection and recording of anthropometric measurements and raise awareness of the significance of these measurements.

The WfLH chart illustrates the correlation between body weight and length in relation to the median (0-line). When WfLH is below the -2SD line on the RTHB graph, it means wasting (Department of Health 2012). Wasting typically occurs because of acute malnutrition, which occurs because of insufficient food intake, inadequate feeding practices, and/or infections. Early identification and intervention can quickly rectify acute malnutrition (Faber 2019). Mid-upper arm circumference serves as one of the indicators of acute malnutrition (MUAC < 12.5 cm) and is considered a reliable screening tool and predictor of mortality (Dukhi et al. 2017). As MUAC is considered a critical indicator, failure to measure it can lead to a missed diagnosis of malnutrition. The anthropometric evaluation of children in the RTHB allows guardians and healthcare providers to monitor children’s growth, improve clinic attendance, and facilitate early identification of growth restrictions (Kitenge 2011). Blaauw et al. (2017) conducted a study evaluating the use of a child health monitoring tool in the Western Cape province, examining RTHBs, and found that weight was plotted more frequently than other indicators such as MUAC, length, or height. Furthermore, the same study revealed that WfA was the most frequently plotted indicator, while WfLH was omitted in more than 50% of RTHBs. Koetaan et al. (2018) reported the recording of WfA in the majority of the RTHBs in the Mangaung Metropolitan Municipality. Another study conducted in Zimbabwe reported that height was not recorded on most children’s growth monitoring cards, only 7% were completed (Marume et al. 2019). These findings raise concerns, as the detection of developmental problems in young children would be challenging. One possible explanation for the lack of height measurements being plotted may be the non-utilisation of length or height measuring tools in healthcare facilities.

Failure to utilise RTHB exposes children to preventable and modifiable diseases such as malnutrition or obesity, which may only be identified at a later stage, making treatment more difficult or even impossible and leading to premature deaths (Bilal et al. 2014). Therefore, the objective of this study is to assess whether the capture of growth indices was performed on the RTHBs in Mangaung Metropolitan Municipality clinics, Free State province.

## Research methods and design

A cross-sectional, quantitative study was conducted on data collected from RTHB of all children who visited primary healthcare (PHC) clinics in the Mangaung Metropolitan Municipality. This approach allowed the researcher to gather information on various variables in the RTHB at a specific moment in time. The clinics were chosen in descending order based on the annual District Health Information System (DHIS) headcount of children under five years of age, ensuring representation from each subdistrict. The study included all RTHBs from 26 clinics, consisting of 6 in Thaba ‘Nchu, 9 in Botshabelo, and 11 in Bloemfontein (Table 1). Road to health booklets were obtained from caregivers of children aged 6–60 months who attended clinics in Mangaung Metropolitan Municipality and willingly agreed to participate in the study.

**TABLE 1:** Distribution of clinics and the road to health booklets per subdistrict.

Sub-district	Number of clinics	Number of RTHBs
Bloemfontein	11	112
Botshabelo	9	120
Thaba ‘Nchu	6	32
**Total**	**26**	**264**

RTHB, road to health booklets.

### Setting

The study was carried out in the Mangaung Metropolitan Municipality, located in the central interior of the Free State province. Previously, this municipality consisted of three subdistricts, namely Bloemfontein, Botshabelo, and Thaba ‘Nchu. Socioeconomically, the district is classified in Quintile 5, which places the Mangaung Metropolitan Municipality among the most affluent districts according to the 2013–2014 District Barometer. The Mangaung Metropolitan Municipality is home to a total of 41 PHC clinics, with the distribution among the three subdistricts as follows: Thaba ‘Nchu has 12 clinics, Botshabelo 12 clinics, and Bloemfontein 17 clinics. The investigation focussed specifically on 26 clinics within the Mangaung Metropolitan Municipality. Each PHC clinic is staffed with one or two professional nurses who are responsible for providing child health services, while most professional nurses in a clinic primarily attend to patients over the age of 5 years. Of the clinics included in the study, 18 were in urban areas, 5 in peri-urban areas, 2 in rural areas, and 1 was a farm clinic.

### Study population and sampling strategy

A non-probability convenience sample was employed to procure RTHBs from caregivers when they brought children for consultation at the clinic. Caregivers were recruited by the researcher in the waiting area of the clinic. Information was collected from 246 RTHBs acquired from caregivers. To maintain confidentiality, each caregiver whose children’s RTHBs were sampled received an exclusive study number.

### Data collection

Data collection was carried out by the researcher and a research assistant between February and August 2018. To collect data, a checklist was used. This checklist was utilised to obtain data that were extracted from the RTHBs. The checklist as an audit tool contained inquiries pertaining to the use of the RTHB aimed at verifying the recording of anthropometric measurements (weigh, length or height, and MUAC), as well as plotting graphs WfA, LHfA, and WfLH on the RTHBs. The 2012 RTHB guideline requires healthcare providers to record weight and length or height measurements taken and plotted monthly during the first year and bimonthly during the second year. Additionally, these measurements could be performed monthly from birth until the child reaches the age of 2 years. According to the RTHB guidelines, MUAC measurements must be performed at least once every 3 months from the age of 6 months to 5 years.

The purpose of the study was explained to caregivers, and caregivers received an information sheet. Caregivers who consented to their child’s RTHB being included in the study were required to sign a consent form. The extraction of data from 264 RTHBs obtained from the caregivers was carried out in a private room after obtaining consent.

### Data analysis

The data from the checklists were entered into a Microsoft Excel spreadsheet and analysed using STATA software version 17 (StataCorp, LLC, College Station, Texas, United States). Descriptive statistics were used to summarise categorical and numeric data.

### Ethical considerations

The study was carried out according to the Declaration of Helsinki and the protocol was approved by the Sefako Makgatho Research Ethics Committee (SMUREC/H/222/2017:PG). All respondents gave their informed consent to include children’s RTHBs in the study.

## Results

### Sociodemographic characteristics

A total of 264 RTHBs were included in the study, of which 51.9% (*n* = 137) belonged to girls and 48.1% (*n* = 127) to boys. The age of the children ranged from 6 months to 60 months with a mean age of 22.7 (+15.30 SD) months. Most of the children whose RTHBs were sampled were between the ages of 6 and 24 months and 36.4% (*n* = 96) were between the ages of 25 and 59 months.

### Recording of anthropometric values

Table 2 illustrates the recording of anthropometric measurements (birth weight, birth length or height, and weight, length or height and MUAC) during last clinic visit recorded in the RTHB. The findings show that 99% (*n* = 262) of the RTHBs had birth weight and 98% (*n* = 260) had birth length recorded. Length and MUAC were not recorded in more than 50% of the RTHBs on the last visit.

**TABLE 2:** Recording of weight, length or height, and mid-upper arm circumference.

Variables recorded	Frequency			
	Yes		No	
	*n*	%	*n*	%
Birth weight	262	99	2	1
Birth length	260	98	4	2
Last weight	252	85	12	5
Last length or height	117	44	147	56
Last MUAC	111	42	153	58

MUAC, mid-upper arm circumference.

**FIGURE 1:**
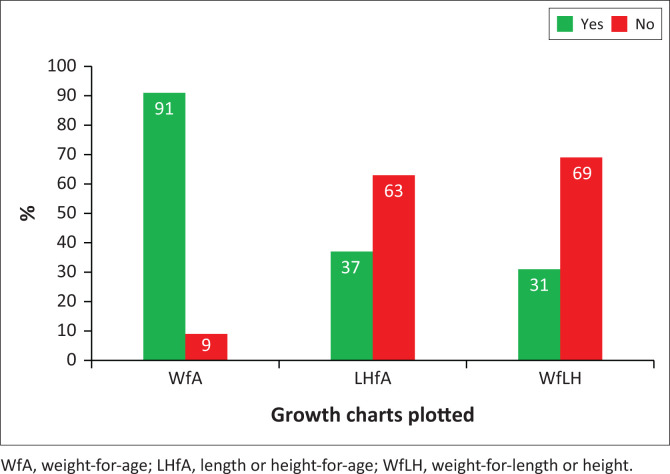
Plotting of weight-for-age, length or height-for-age and weight-for-length or height.

The findings in Figure 1 show good practice during the last clinic visit, where 91% (*n* = 241) of the RTHBs had the WfA plotted on the growth chart. The LHfA was not plotted in 63% (*n* = 165) of the RTHBs and was plotted in 37% (*n* = 99) of the RTHBs. The plotting of WfLH was performed in 31% (*n* = 81) of the RTHBs and not in 69% (*n* = 183) of the RTHBs.

### Number of road to health booklets with recording of all indices

**FIGURE 2:**
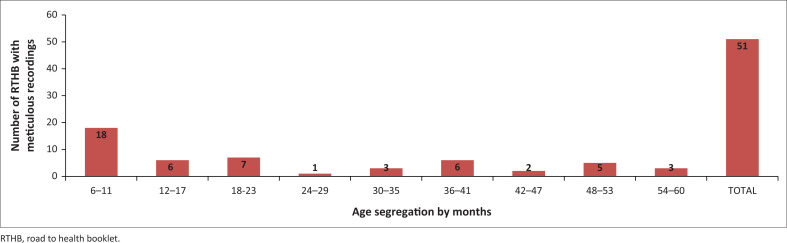
Number of road to health booklet with meticulous recording of all indices by age segregation.

Figure 2 shows 51 RTHBs per age segregation in months that were fully completed with all indices, but did not include nutritional classifications (wasting, overweight, stunting, or underweight). Good practice was observed with respect to fully completed indices for RTHBs of children aged 6 to 11 months 35% (*n* = 18), and fewer completed RTHBs were observed for children aged 48–53 months 10% (*n* = 5), 30–35 months 6% (*n* = 3), 54–60 months 4% (*n* = 2), and 2% (*n* = 1) at the age of 24–29 months.

The data presented in Figure 1 and Figure 2 indicated that 19% of RTHBs were meticulously completed for ages 6 to 60 months. Figure 1 showed that 91% (*n* = 241) WfA, 37% (*n* = 99) LHfA, and 31% (*n* = 81) WfLH were plotted, which was likely to be in children of the age group between 6 and 11 months, constituting 35% RTHBs of meticulously recorded all the indices as illustrated in Figure 2.

## Discussion

The primary objective of the study was to assess whether the capture of the growth indices on the RTHBs is performed in Mangaung Metropolitan Municipality clinics of the Free State province. The sampled records included RTHB data from children with a mean age of 23 months.

The findings of this study revealed that birth weight and birth duration were recorded in almost all RTHBs, which is considered a customary practise to document all anthropometric variables immediately after birth. However, in the RTHBs of children older than six months of age during their last clinic visits, less than half of the records contained information on their length and upper arm circumference (MUAC). The number of RTHBs with almost complete recordings decreased as the child grew older. These findings are in line with what has been reported by McLaren, Steenkamp and Ronaasen (2024) in their study that older children did not have complete growth indices measured.

Mid-upper arm circumference was not recorded in most RTHBs. A similar study conducted by Blaauw et al. (2017) in the Western Cape province, examining the utilisation of a child health monitoring tool, reported that anthropometry was performed mainly for weight and minimally for both MUAC and length or height. Koetaan et al. (2018) also found inadequate recording of MUAC in the RTHBs, suggesting that MUAC measurement is not commonly performed in most children. Incomplete recording of anthropometric indicators negatively affects the overall assessment of nutrition because values are not available to plot on RTHB graphs (Marume et al. 2019). Mid-upper arm circumference values are essential for the evaluation of nutritional status at the primary care level.

Most RTHBs had WfA measurements plotted, indicating that it is common practice in most clinics. The findings of Blaauw et al. (2017) also showed a similar pattern of WfA recordings in the majority of RTHBs. Apparently, the main reason for the high prevalence of WfA measurements in the growth chart is that healthcare providers perceive it as the primary determinant of a child’s nutritional status (Koetaan et al. 2018).

The study’s findings show that LHfA and WfLH were the least plotted indicators. Koetaan et al. (2018) also reported similar findings, indicating insufficient plots of LHfA and WfLH in the growth chart. The study results by Blaauw et al. (2017) in the Western Cape showed that in more than half of the RTHBs, LHfA was plotted and less than half of the booklets had WfLH plotted. The results on the plotting of LHfA and WfLH in the Western Cape were higher than those found in this study as well as in a study conducted by Koetaan et al. (2018) in the Free State. The higher percentage of plotting LHfA and WfLH in the Western Cape may be because of better training or a higher number of healthcare personnel. The findings of the study by Marume et al. (2019) in Zimbabwe concur with the findings of this study in that length or height of the children were not measured and LHfA was only plotted in a few of the RTHBs of children under five years of age who visited the clinic. The practice in clinics of not plotting LHfA and WfLH typically results in the child health nurse not being able to assess the nutritional status of children adequately, which is required for nutrition classification and interpretation (Dimo, Madiba & Bhayat 2023).

Non-compliance with the child health guidelines on recording, plotting, and interpreting the nutritional status of children as part of vital health screening challenges the ability to report accurately and delays diagnosis and treatment of acute malnutrition, overweight, and stunting. Poor record keeping also affects the ability to accurately report on SDG-2 and global nutrition goals.

### Limitation of the study

The checklist for auditing RTHBs should have asked caregivers if they understand the importance of growth monitoring with the aim of increasing service demand regarding growth monitoring in the consulting room and verifying routine use of anthropometric equipment, recording values, plotting graphs and noting nutrition classification in the RTHB.

### Recommendations

Health care providers in accordance with the guidelines must provide information on GMP to caregivers to understand the importance of recording all the nutritional indices in RTHB.

## Conclusion

This study revealed that some of the indices that are critical for nutrition classification were not captured in the RTHBs. The recording was mainly based on weight measurement and plotting of WfA and did not consider length and MUAC. Therefore, very few RTHBs plotted LHfA and WfLH graphs. Consequently, RTHBs lack notes on nutrition classification of children reported as normal, underweight, wasted, overweight, and stunted.

Primary healthcare managers should reinforce growth monitoring as a compulsory practice of vital health screening according to the RTHB guidelines.

The National Department of Health should review national indicator data sets, which should include reporting incidence of nutritional classification as normal, overweight, wasted, overweight and stunted in the District Health Information System.
